# Prevalence of goiter and associated factors among schoolchildren in northeast Ethiopia

**DOI:** 10.4178/epih.e2017055

**Published:** 2017-11-25

**Authors:** Emebet Tigabu, Kindie Bantie Bekele, Berihun Assefa Dachew

**Affiliations:** 1 Ethiopian Paediatric Association, Amhara Region, Bahir Dar, Ethiopia; 2 Department of Epidemiology and Biostatistics, Institute of Public Health, College of Medicine and Health Sciences, University of Gondar, Gondar, Ethiopia

**Keywords:** Goiter, Prevalence, Risk factors, Ethiopia

## Abstract

**OBJECTIVES:**

Goiter is a major public health problem, especially in developing countries such as Ethiopia. Hence, this study aimed to assess the prevalence and associated factors of goiter among children in Waghimra Zone, northeast Ethiopia.

**METHODS:**

A cross-sectional study was conducted from April 8 to 25, 2015 in northeast Ethiopia. A multistage sampling method was used to select 454 schoolchildren. Data were collected using a pre-tested structured interviewer-administered questionnaire. Children were examined for the presence or absence of goiter based on the criteria of the United Nations Children’s Fund, International Council for the Control of Iodine Deficiency, and the World Health Organization. Salt samples from children’s homes were tested for iodine levels using a rapid iodized salt test kit. Data were entered into EpiInfo version 7 and exported to SPSS version 20 for analysis. Bivariate and multivariate logistic regression models were fitted, and adjusted odds ratio (aOR) with 95% confidence interval (CI) were computed to determine the level of significance.

**RESULTS:**

The prevalence of goiter was 62.1% (95% CI, 57.5 to 66.5%). Being female (aOR, 3.09; 95% CI, 1.57 to 6.08), having a family history of goiter (aOR, 5.18; 95% CI, 2.43 to 11.03), and using non-iodized salt (aOR, 2.20; 95% CI, 1.12 to 4.38) were factors associated with goiter among schoolchildren.

**CONCLUSIONS:**

The prevalence of goiter was high. Being female and having a family history of goiter increased the risk of goiter in children, but using iodized salt was protective. Therefore, we recommend ensuring universal access to iodized salt and increasing the awareness of the community of the importance of iodized salt utilization.

## INTRODUCTION

Iodine is one of the essential micronutrient components of the thyroid hormones (thyroxin and triiodothyroxine), which regulate physical and mental development in children [1]. Low blood levels of iodine lead to an insufficient production of these hormones in the body, resulting in iodine deficiency disorder (IDD) [1].

Globally, 30% of the world’s population is affected by IDD [2,3]. Goiter, an indicator of chronic iodine deficiency, is a major public health problem in several areas of the world [4]. A study based on the World Health Organization (WHO) global database showed that the total goiter prevalence (TGP) of Africa was 28.3% [5].

Ethiopia is among the most iodine-deficient countries in the world [6]. A nationwide study conducted in Ethiopia revealed that the TGP in schoolchildren was 39.9% [7]. However, IDD is the easiest and cheapest of all disorders to prevent. The most effective solution to this problem is the application of universal salt iodization [3,8], which is the most effective, low-cost, and long-term solution that should be deployed on a daily basis, especially in iodine-deficient environments [2]. Ethiopia endorsed a universal salt iodization program with the goal of reaching more than 90% coverage [9]. An Ethiopian Public Health Institute report found that over 88% of the salt in the country contained iodine, but only 23.2% of the households used adequately iodized salt [10].

School-age children are the preferred group for inspection and palpation for goiter and IDD surveillance because of their high vulnerability, ease of access, and the high effectiveness of the treatment in this population. Additionally, their iodine status is a proxy for the iodine status of the general population. Various studies have indicated that the prevalence of goiter in children is an indicator of local iodine consumption and is a signal of to what extent the community is affected by the disorder [3-5]. Therefore, investigating the prevalence of goiter is of paramount significance, although there is a dearth of literature on this topic, particularly in the area analyzed in this study. Thus, this study aimed to determine the prevalence of goiter and associated factors among school-age children in Waghimra Zone, northeast Ethiopia.

## MATERIALS AND METHODS

This school-based cross-sectional study was conducted from April 8 to 25, 2015 among children aged 7-12 years in Gasgibla *woreda*, Waghimra Zone, northeast Ethiopia. This *woreda* is located 755 km from Addis Ababa and 561 km from the regional capital of Bahir Dar.

The sample size was determined by a single-population proportion formula using the EpiInfo program (Centers for Disease Control and Prevention, Atlanta, GA, USA) using the following assumptions: population size, 95% confidence interval (CI), 5% marginal error, and 59.1% prevalence of goiter (which was taken from another similar study) [11]. Considering a 5% non-response rate and a design effect of 1.5, the required sample size was 454. Multistage sampling followed by a systematic random sampling technique was employed to select study participants. Out of the government-owned primary schools in the study area, 4 (20%) were selected by simple random sampling. By using the student registration list as a sampling frame, systematic random sampling was applied to select participants from each school.

An interviewer-administered structured questionnaire was used to collect the data. The questionnaire was first prepared in English, translated into the local language (Amharic) and finally, back-translated to English to maintain consistency. Two emergency surgeons (for the physical examination) and 4 nurses were recruited to collect the data. Only the physical examination was directly done with each child, while the rest of the information was collected from the mother/caregiver of the child. A day before data collection, the name, age, sex, and residence of the students were identified from the school register.

A physical examination was performed at school to identify the presence and absence of goiter in accordance with the criteria of the United Nations Children’s Fund, International Council for the Control of Iodine Deficiency, and the WHO [12]. The child’s neck was inspected and then palpated for abnormal masses. Goiter was considered to be absent if no palpable or visible goiter was present (grade 0), whereas goiter was considered to be present when a child had grade 1 or grade 2 goiter, or both. Data on dietary habits and major goitrogenic food items in the locality, such as millet, cow milk, cabbage, and sorghum, were also collected.

On the day of the physical examination, the children included in the study were instructed to bring a pinch of the salt used at their home. The iodine content of the sampled salt was estimated using a rapid iodized salt test kit (MBI test kit; MBI Kits International, Tamil Nadu, India). The kit contained a stabilized starch-based solution that causes a chemical reaction manifested by a color change. The salt sample was taken in a teaspoon, and a drop of the test solution was poured on the salt [13]. Salt samples with less than 15 parts per million (ppm) of iodine content were classified as having inadequate iodine [14].

After checking for completeness, data were entered into EpiInfo version 7 (Centers for Disease Control and Prevention) and exported to SPSS version 20 (IBM Corp., Armonk, NY, USA) for analysis. Descriptive statistics, including frequencies and proportions, were used to summarize the variables. Both bivariate and multivariate logistic regression analyses were performed. A *p*-value of 0.2 was used as a screening criterion for including variables in the multivariable model. In the multivariate analysis, a *p*-value of ≤ 0.05 was considered to indicate statistical significance. The variables were entered into the multivariate model using the forward stepwise regression method.

Ethical clearance was obtained from the institutional review board of the Bahir Dar University Institute of Technology. An official permission letter was secured from the Amhara Regional Health Bureau Research Centre. The confidentiality of the information was maintained. Written or verbal (for those who could not read or write) consent was obtained from each mother/guardian. Assent was obtained from children before palpating their thyroid gland. Children who were found to have goiter were referred to a health institution to receive appropriate treatment and support.

## RESULTS

### Socio-demographic characteristics

A total of 443 children with a mother/caregiver participated in the study, corresponding to a response rate of 97.6%. More than half (54.0%) of them were females, with a mean age of 9.77± 1.74 years. Regarding the socio-demographic characteristics of the mothers/caregivers, the majority were married (78.8%) and 38.1% were farmers by occupation. Table 1 shows the socio-demographic characteristics of the children and their mothers /caregivers in the study area.

### Dietary habit and goitrogenic food consumption by children

Most of the children (89.6%) had a history of eating sorghum frequently; 76.0% of them consumed it daily. The second most commonly consumed goitrogenic staple food was millet, which was reported to be consumed more than once per month by 101 of the children (22.8%). Among vegetables and dairy products, cabbage and milk were consumed by 347 (78.3%) and 170 (38.4%) of the children, respectively, and rivers were the source of drinking water for 26.9% of the children (Table 2).

### Knowledge and utilization of iodized salt by mothers

In this study, 245 mothers (55.3%) were aware of iodized salt, but only 150 (61.2%) used iodized salt. The common reasons mentioned by the respondents for not using iodized salt were unavailability and cost, which were reported by 54 (56.8%) and 37 (39.0%) of them, respectively. Only 17.2% of the households used salt with an adequate iodine level (≥ 15 ppm) (Table 3).

### Prevalence of goiter and factors independently associated with goiter

The overall prevalence of goiter in schoolchildren (7-12 years) was found to be 62.1% (95% CI, 57.5 to 66.5%). Logistic regression analysis was carried out for socio-demographic and other characteristics, including age, sex, dietary habits, knowledge of iodized salt, and its consumption at the household level. In the bivariate logistic regression, the child’s age, sex, place of birth, iodized salt utilization, and family history of goiter were associated with goiter. However, in the final multivariate logistic regression, only the child’s sex, family history of goiter, and iodized salt utilization were found to be significantly associated with our outcome variable. The probability of developing goiter was about 3 times higher among females (adjusted odds ratio [aOR], 3.09; 95% CI, 1.57 to 6.08) than among males. Children from households that did not use iodized salt were about 2 times more likely to have goiter (aOR, 2.20; 95% CI, 1.12 to 4.38) than their counterparts. The likelihood of developing goiter was about 5 times greater (aOR, 5.18; 95% CI, 2.43 to 11.03) among children with a family history of goiter than among children without a family history of goiter (Table 4).

## DISCUSSION

In this study, the prevalence of goiter was determined to be 62.1%. This rate is much higher than the cut-off point of the WHO IDD classification (5.0%) [4]. This finding is also higher than those of other studies. For example, the Ethiopian national average prevalence was found to be 39.9% [7], while that of Gondar and Jimma was 37.6% [15] and 59.1% [11], respectively. A possible reason for the variations from place to place could be the density of grass. Our study was conducted in an over-grazed and mountainous environment highly exposed to soil erosion, which washes away the iodine in the topsoil, resulting in iodine-deficient food crops and leading to a low level of iodine content in the staple foods of the inhabitants. However, the prevalence we observed is lower than the finding reported in a study conducted in Tigray (71.4%) [16]. This may be because our study was undertaken after universal salt iodization was launched in Ethiopia.

This study showed that the non-utilization of iodized salt in a household was associated with the development of goiter in children. Non-iodized salt users were about 2 times more likely to have goiter than iodized salt users. This finding is in agreement with the findings of other similar studies [5,11,17]. This could be because iodized salt has both preventive and corrective effects for iodine deficiency goiter, and is the main solution for eradicating IDD. The WHO has recommended that 90% of the households in a population should be able to obtain iodized salt with an iodine level > 15 ppm for the effective elimination of IDD [4]. However, in this study, only 76 households (17.2%) had adequate salt, with an iodine level > 15 ppm. This may have been a primary reason for the high prevalence of goiter in the study area. The low coverage of iodized salt, coupled with a low awareness of the importance of iodized salt, and its high cost are likely the factors that are fueling the high prevalence of goiter.

The prevalence of goiter was higher among females in our study. This finding is consistent with reports from various studies conducted throughout the world [11,16-18]. This could be due to the physiological differences between males and females. That is, females start spurt growth earlier than males and require more iodine.

Our study demonstrated a significant association between family history and goiter. Children whose parents had goiter had about a 5-fold increased risk of developing goiter when compared to those who had no family history. This finding is similar to those of other studies [11,15], and could be due to the fact that malnutrition has inter-generational cycles [19].

The study has some limitations. Since only schoolchildren were included in this study, the results may not be generalizable to children who are not in school. In addition, there may have been a misclassification bias in the determination of salt iodine levels by the rapid test kit, as other laboratory tests, such as a urinary iodine excretion test, blood tests for thyroid-stimulating hormone, and testing of the iodine levels of water were not done. Finally, although we asked the mothers/caregivers during the interview period to ascertain that the salt brought by the children was truly from their home, we were not able to fully exclude this potential problem.

In summary, the overall prevalence of goiter was found to be high in the present study. In this study, being female and having a family history of goiter were associated with an increased risk of goiter in children. In contrast, using iodized salt was found to be a protective factor against goiter. The risks and consequences of IDD should be emphasized, and programs aimed at the prevention and control of IDD should gain attention at all levels. Ensuring universal accessibility and availability of iodized salt at the household level is strongly recommended. Further research with a better methodological design, including laboratory methods to determine the iodine content of water and urinary iodine excretion, is recommended.

## Figures and Tables

**Table 1.** Socio-demographic characteristics of children and their mothers/caregivers in Gasgibla *woreda*, Waghimra Zone, northeast Ethiopia, 2015

Variables	n (%)
Child characteristics	
Age (yr)	
7-9	192 (43.3)
10-12	251 (56.7)
Sex	
Female	239 (54.0)
Male	204 (46.0)
Characteristics of mothers/caregivers	
Marital status	
Married	349 (78.8)
Divorced	40 (9.0)
Single	34 (7.7)
Widowed	20 (4.5)
Educational status	
Cannot read and write	225 (50.8)
Read and write	164 (37.0)
Elementary	35 (7.9)
Secondary	13 (2.9)
Above secondary	6 (1.3)
Occupational status	
Housewife	193 (43.6)
Farmer	169 (38.1)
Merchant	49 (11.1)
Civil servant	10 (2.3)
Others^1^	22 (5.0)

1 Daily laborer, private worker.

**Table 2.** Dietary habit and goitrogenic food consumption of children aged 7-12 years in Gasgibla *woreda*, Waghimra Zone, northeast Ethiopia, 2015

Variables	Frequency (%)
Sorghum consumption (n=388)	
At least once per day	329 (76.0)
At least once per week	32 (7.4)
At least once per month	27 (6.2)
Millet consumption (n=101)	
At least once per day	32 (7.2)
At least once per week	10 (2.3)
At least once per month	59 (13.3)
Cabbage consumption (n=347)	
At least once per day	31 (7.0)
At least once per week	187 (42.2)
At least once per month	129 (29.1)
Milk consumption (n=170)	
At least once per day	19 (4.3)
At least once per week	100 (22.6)
At least once per month	51 (11.5)
Source of drinking water (n=433)	
Pipe	324 (73.1)
River	119 (26.9)

**Table 3.** Knowledge and utilization of iodized salt by mothers in Gasgibla *woreda*, Waghimra Zone, northeast Ethiopia, 2015

Variables	n (%)
Do you know about iodized salt?	
Yes	245 (55.3)
No	198 (44.7)
If yes, do you use it?	
Yes	150 (61.2)
No	95 (38.8)
If yes, when do you add iodized salt?	
During cooking	81 (54.0)
After cooking	65 (43.3)
Cannot remember	4 (2.7)
Reason for not using iodized salt	
Lack of access	54 (56.8)
Cost	37 (39.0)
Other^1^	4 (4.2)
Iodine level of salt (ppm)	
0	269 (60.7)
<15	98 (22.1)
15-30	76 (17.2)

ppm, parts per million.

1 No difference, not tasty.

**Table 4.** Factors associated with goiter among schoolchildren, Gasgibla *woreda*, Waghimra Zone, northeast Ethiopia, 2015

Variables	Goiter		OR (95% CI)	aOR (95% CI)^1^
	Yes	No		
Age (yr)				
7-9	104	88	1.00 (reference)	
10-12	171	80	1.81(1.23, 2.67)	
Sex				
Male	103	101	1.00 (reference)	1.00 (reference)
Female	172	67	2.52 (1.67, 3.73)	3.09 (1.57, 6.08)^*^
Place of birth				
Zarota	100	46	1.00 (reference)	
Asketema	57	61	1.02 (0.59, 1.76)	
Kieta	47	29	2.37 (1.37, 4.12)	
Bela	71	32	1.37 (0.73, 2.53)	
Iodized salt utilization				
Yes	81	69	1.00 (reference)	1.00 (reference)
No	194	99	1.67 (1.07, 3.16)	2.20 (1.12, 4.38)^*^
Family history of goiter				
Yes	190	72	2.98 (2.01, 4.44)	5.18 (2.43, 11.03)^*^
No	85	89	1.00 (reference)	1.00 (reference)

OR, odds ratio; CI, confidence interval; aOR, adjusted OR.

1 The forward stepwise regression method was used.

* p<0.05.
